# Mathematical Modelling of Canola Oil Biodegradation and Optimisation of Biosurfactant Production by an Antarctic Bacterial Consortium Using Response Surface Methodology

**DOI:** 10.3390/foods10112801

**Published:** 2021-11-14

**Authors:** Khadijah Nabilah Mohd Zahri, Khalilah Abdul Khalil, Claudio Gomez-Fuentes, Azham Zulkharnain, Suriana Sabri, Peter Convey, Sooa Lim, Siti Aqlima Ahmad

**Affiliations:** 1 Department of Biochemistry, Faculty of Biotechnology and Biomolecular Sciences, Universiti Putra Malaysia, Serdang 43400, Selangor, Malaysia; khadijahnabilah95@gmail.com; 2 School of Biology, Faculty of Applied Sciences, Universiti Teknologi MARA, Section 2, Shah Alam 45000, Selangor, Malaysia; khali552@uitm.edu.my; 3 Department of Chemical Engineering, Universidad de Magallanes, Avda. Bulnes 01855, Punta Arenas, Chile; claudio.gomez@umag.cl; 4 Center for Research and Antarctic Environmental Monitoring (CIMAA), Universidad de Magallanes, Avda. Bulnes 01855, Punta Arenas, Chile; 5 Department of Bioscience and Engineering, College of Systems Engineering and Science, Shibaura Institute of Technology, 307 Fukasaku, Minuma-ku, Saitama 337-8570, Japan; azham@shibaura-it.ac.jp; 6 Department of Microbiology, Faculty of Biotechnology and Biomolecular Sciences, Universiti Putra Malaysia, Serdang 43400, Selangor, Malaysia; suriana@upm.edu.my; 7 British Antarctic Survey, NERC, High Cross, Madingley Road, Cambridge CB3 0ET, UK; pcon@bas.ac.uk; 8 Department of Zoology, University of Johannesburg, P.O. Box 524, Auckland Park 2006, South Africa; 9 Department of Pharmaceutical Engineering, Hoseo University, Asan-si 31499, Chungnam, Korea; salim0609@hoseo.edu

**Keywords:** kinetic modelling, canola oil, degradation, biosurfactant, Antarctic bacteria, central composite design

## Abstract

An Antarctic soil bacterial consortium (reference BS14) was confirmed to biodegrade canola oil, and kinetic studies on this biodegradation were carried out. The purpose of this study was to examine the ability of BS14 to produce biosurfactants during the biodegradation of canola oil. Secondary mathematical equations were chosen for kinetic analyses (Monod, Haldane, Teissier–Edwards, Aiba and Yano models). At the same time, biosurfactant production was confirmed through a preliminary screening test and further optimised using response surface methodology (RSM). Mathematical modelling demonstrated that the best-fitting model was the Haldane model for both waste (WCO) and pure canola oil (PCO) degradation. Kinetic parameters including the maximum degradation rate (*μ_max_*) and maximum concentration of substrate tolerated (*S_m_*) were obtained. For WCO degradation these were 0.365 min^−1^ and 0.308%, respectively, while for PCO they were 0.307 min^−1^ and 0.591%, respectively. The results of all preliminary screenings for biosurfactants were positive. BS14 was able to produce biosurfactant concentrations of up to 13.44 and 14.06 mg/mL in the presence of WCO and PCO, respectively, after optimisation. The optimum values for each factor were determined using a three-dimensional contour plot generated in a central composite design, where a combination of 0.06% salinity, pH 7.30 and 1.55% initial substrate concentration led to the highest biosurfactant production when using WCO. Using PCO, the highest biosurfactant yield was obtained at 0.13% salinity, pH 7.30 and 1.25% initial substrate concentration. This study could help inform the development of large-scale bioremediation applications, not only for the degradation of canola oil but also of other hydrocarbons in the Antarctic by utilising the biosurfactants produced by BS14.

## 1. Introduction

Antarctica is commonly considered the most pristine landmass on Earth, making it the perfect place for monitoring the spread of global pollutants as well as being a sensitive indicator of global climate change [1]. Nevertheless, pollution events such as hydrocarbon oil spills (diesel, petroleum and engine as well as waste oil) have taken place in many parts of the Antarctic, affecting terrestrial, freshwater, marine and ice environments [2]. Shipping has a high potential to pollute seawater through waste oil generated from grey water and food waste, which includes waste cooking oil; for instance, a larger cruise ship carrying 2700 passengers can generate over one tonne per day [3]. Anthropogenic activities are clearly responsible for the release of oil into the environment, causing chronic toxicity and sub-lethal effects in natural ecosystems, often affecting localised areas [4,5,6,7,8].

The potential of microorganisms has been recognized in the development of strategies to remediate oil spills. Specifically relating to the biodegradation of waste cooking oils, Zahri et al. [9] studied the degradation of waste canola oil (WCO) and pure canola oil (PCO) using a consortium of native Antarctic soil bacteria. Microorganisms are increasingly used to treat and transform waste products, and are considered as an eco-friendly technology for the remediation of contaminated environments. Detailed mathematical regression approaches have been applied to model the kinetics of hydrocarbon bioremediation and identify the optimal environmental conditions required [10]. Such kinetic studies allow the determination of the time needed for a contaminant to be degraded to a target concentration and underpin the design of biodegradation kinetics models.

Biosurfactants are a useful tool in bioremediation that can act as emulsion stabilisers (either as an emulsifier or de-emulsifier) and anti-adhesive agents through mobilisation and solubilisation mechanisms [11]. The biosurfactants’ function is derived from their amphiphilic composition, via the water-soluble and water-insoluble elements included in their molecular structure [12]. Biosurfactants have the capability to increase the surface area of hydrocarbons by lowering the interfacial tension between the hydrophilic and hydrophobic parts and, thus, can play a key role in the bioremediation process. The efficiency of bacterial production of biosurfactants can be affected by various nutritional and environmental factors. Factors such as salinity, temperature, pH, type and concentration of substrate can determine the stability of microbial biosurfactants [13]. Thus, biosurfactant production factors must be monitored and maintained within a specific range of operating conditions to achieve the optimum production of biosurfactants.

A native Antarctic soil bacterial consortium, designated BS14, has been previously confirmed to degrade canola oil [9,14]. Further studies on the potential of this consortium are described here, involving degradation kinetics modelling using secondary non-linear regression. The ability of the consortium to produce biosurfactants during the biodegradation of canola oil was also analysed and optimised through the application of response surface methodology (RSM). This research will contribute to the development of bioremediation approaches involving the use of biosurfactants and offering efficient and cost-effective means to treat contaminated areas, thereby improving environmental protection.

## 2. Materials and Methods

Consortium BS14 was originally obtained from soil collected in the vicinity of the Chilean General Bernardo O’Higgins Riquelme research station (Trinity Peninsula, northwest Antarctic Peninsula; 63°19′20.6′′ S 57°53′53.6′′ W). The isolation and preparation of the consortium are described by Zahri et al. [9]. Both waste canola oil (WCO) and pure canola oil (vegetal PCO) (Belmont, Chile) were acquired from the research station’s kitchen. In Antarctica, WCO is stored in steel oil drums at room temperature. There was no specific information available on the number of times the canola oil was repeatedly heated. This is because the uses of canola oil in Antarctic include various type of cooking (frying, grilling and boiling) and types of food prepared.

### 2.1. Shake Flask Culture Conditions

A modified medium from Zahri et al. [9] was used for the biodegradation of canola oil: minimal salt medium (MSM). For study of the degradation of WCO the medium consisted (per L) of 8.34 g K_2_HPO_4_, 2.61 g KH_2_PO_4_, 0.13% *w*/*v* NaCl, 1 g (NH_4_)_2_SO_4_, 1.13 g yeast extract and 1% *v*/*v* WCO with the pH adjusted to 7.13 using HCl. The MSM for PCO consisted (per L) of 9.52 g K_2_HPO_4_, 2.00 g KH_2_PO_4_, 0.75 g (NH_4_)_2_SO_4_, 1.00 g yeast extract and 1% PCO, with the pH adjusted to pH 7.25 using HCl. The bacterial consortium culture (1 mL) was inoculated into 50 mL of both MSM media and incubation took place on an orbital shaker (150 rpm) at 10 °C.

### 2.2. Biodegradation Kinetics Modelling

The specific degradation rate with different initial substrate concentrations of WCO and PCO was obtained through assessment of the percentage of degradation each day over the 7 days incubation period. The fit of the data obtained to different widely used non-linear regression models was then assessed, including Monod, Haldane, Teissier–Edwards, Aiba and Yano models [15,16,17,18,19]. Varying environmental conditions have impacts on the performance of each kinetic model. Important kinetic parameters can be obtained through these established regression models, including the maximum degradation rate (*μ_max_*), half-saturation constant (*K_s_*), inhibition constant (*K_i_*) and maximum initial concentration of substrate tolerated (*S_m_*). The data were fitted using CurveExpert Professional software (version 2.6, Hyams Development, Huntsville, AL, USA).

#### Percentage Degradation of WCO and PCO

The amount of residual canola oil after biodegradation was determined gravimetrically after day 7 [20]. The residual oil was extracted using n-hexane organic solvent in a 1:1 ratio with the media in a 100 mL separating funnel. The mixture was vigorously shaken and then left to stand for 15 min to allow separation to occur. The aqueous layer was removed, and the organic layer was transferred into a Petri dish and concentrated by evaporation in a fume hood for 24 h. The mass of residual oil was determined and corrected by taking into consideration the residual oil from the abiotic control. The percentage biodegradation of WCO and PCO was calculated using Equation (1) [21]:

(1) $$\mathrm{Biodegradation}\;\left(\%\right)=\left(\frac{\begin{array}{c} Weight\;of\;residual\;canola\;oil\;\left(abiotic\;control\right)-\\ weight\;of\;residual\;canola\;oil\;\left(sample\right) \end{array}}{Original\;weight\;of\;introduced\;canola\;oil}\right)\times 100$$

### 2.3. Analysis of Biosurfactant Production

The ability of BS14 to produce biosurfactants was screened by assessing haemolytic activity, microbial adhesion to hydrocarbons (MATH) assay, drop-collapse test, oil displacement test and emulsification index (E_24_). A separate Antarctic bacterial consortium (BS5) known to not produce biosurfactants was used as the negative control in each assay. BS5 was also obtained from the vicinity of the research station.

#### 2.3.1. Haemolytic Activity

Blood agar plates were used to screen for biosurfactant activity. BS14 cultured on MSM media (incubated for 2 days) were inoculated onto blood agar plates containing Columbia agar with 5% sheep blood and incubated for 5–6 days at 10 °C. The presence of a clear zone around the colonies confirmed the presence of biosurfactant synthesis by the bacteria [22].

#### 2.3.2. Microbial Adhesion to Hydrocarbons (MATH) Assay

One-day-old BS14 cultures grown on both MSM media were harvested by centrifugation at 5000 rpm for 20 min at 4 °C, washed and rinsed twice with 1× phosphate-buffered saline (PBS). The cells were suspended for standardisation at 1 ± 0.01 OD_600 nm_ using PBS. Then, 300 μL of the organic solvents hexadecane and tetrahexadecane was added to 5 mL of bacterial cell suspension in separate test tubes. The mixtures in the test tubes were vortexed for 2 min and then left to stand for 15 min to allow for the separation of the solvent and medium [23]. The aqueous phase was carefully removed from the tubes, and its absorbance was measured at 600 nm using a spectrophotometer. Microbial adhesion to hydrocarbons was calculated using Equation (2):

(2) $$\mathrm{MATH}\;\left(\%\right)=\left[1-\left(\frac{\mathrm{Final}\;\mathrm{absorbance}\;\mathrm{reading}\;\left(\mathrm{nm}\right)}{\mathrm{Initial}\;\mathrm{absorbance}\;\mathrm{reading}\;\left(\mathrm{nm}\right)}\right)\right]\times 100$$

#### 2.3.3. Drop-Collapse Test and Oil Displacement

This method was modified from Rani et al. [24]. MSM BS14 grown cultures (7 days) containing WCO or PCO were centrifuged at 5000 rpm for 20 min at 4 °C to obtain cell-free supernatants. Then, about 20 μL of used engine oil (obtained from a motorcycle repair shop in Sri Serdang, Selangor, Malaysia) was set on a grease-free glass slide followed by the addition of 50 μL of the cell-free supernatant onto the centre of the engine oil. Flattening of the oil drop is considered a positive indication of biosurfactant production.

Oil displacement activity was assessed using 2 mL of used engine oil added to a Petri dish containing 50 mL of sterile H_2_O. Then, 100 μL of cell-free WCO or PCO MSM culture supernatants was carefully added to the engine oil surface and the diameter of the clear zone on the oil surface was measured [25].

#### 2.3.4. Emulsification Index (E24)

Three millilitres of cell-free WCO or PCO MSM culture supernatant (grown for 7 days) was added to an equal amount of different types of hydrocarbons (commercial canola oil and toluene) in separate test tubes. This included WCO and PCO that consisted of frying canola oil, 100% canola oil and canola vegetable oil (Belmont, Chile). The tubes were then thoroughly mixed by vortexing for 5 min. The height of the emulsion layer was measured after 24 h. The emulsification activity index E_24_ was calculated using Equation (3) [26]:

(3) $$E24\;\left(\%\right)=\left(\frac{\mathrm{Height}\;\mathrm{of}\;\mathrm{emulsification}\;\left(\mathrm{cm}\right)}{\mathrm{Total}\;\mathrm{height}\;\mathrm{of}\;\mathrm{the}\;\mathrm{mixture}\;\left(\mathrm{cm}\right)}\right)\times 100$$

All commercialised PCO oil consisted of different compositions of oil, where (1) frying canola oil was composed of high oleic canola oil, high oleic wonder oil, corn oil, canola oil, vitamin E, citric acid and dimethylpolysiloxane, (2) 100% canola oil was composed of canola oil, vitamin E, citric acid as well as dimethylpolysiloxane and (3) canola vegetable oil was composed of soybean oil (90%), canola oil (10%), citric acid and dimethylpolysiloxane.

### 2.4. Extraction of Biosurfactants

To extract the biosurfactants produced, the cell-free WCO and PCO MSM culture supernatants were first centrifuged at 5000 rpm for 20 min at 4 °C and then filtered through a Whatman No.1 filter paper. Then, any remaining WCO or PCO in the supernatant was removed before the extraction process, and the pH was adjusted to pH 2 using 2N HCl. Subsequently, a 2:1 ratio of chloroform:methanol solution was added to the test tubes. The mixtures were vigorously vortexed for 15 min to allow a separation process to occur [27]. The organic phase (chloroform + biosurfactant) was placed in glass Petri dishes and dried in an oven at 80 °C for 1 day to completely evaporate the solvent. After drying, the glass Petri dishes were weighed, and the dry mass of the biosurfactants was calculated using Equation (4) [28]:

(4) $$\mathrm{Biosurfactant}\;\mathrm{mass}\;\left(\mathrm{mg}\right)=\mathrm{Mass}\;\mathrm{of}\;\mathrm{plate}\;\mathrm{after}\;\mathrm{drying}\;-\mathrm{Mass}\;\mathrm{of}\;\mathrm{the}\;\mathrm{empty}\;\mathrm{plate}$$

### 2.5. Experimental Design and Optimisation

The production of biosurfactant was screened daily for 10 days to determine the maximum biosurfactant production period. Then, the BS14 consortium was cultured under different conditions of salinity, pH, temperature and initial substrate concentration for the optimisation process with the incubation time at the highest production of biosurfactant during the screening process.

The Plackett–Burman design (PBD) was used to screen for the critical factors and eliminate all non-significant factors for further optimisation in the central composite design (CCD). The design did not designate the interaction between the factors [29]. Twelve experimental runs were generated where each experiment contained two factorials: low (1) and high (+) levels for each factor (Table 1). The range of low and high levels was based on our previous study, optimising bacterial growth and biodegradation of WCO and PCO by BS14 [9].

Statistical optimisation of biosurfactant production was carried out using a CCD, which is useful in identifying significant pairwise interactions between factors which affect the optimisation process. CCDs require only a small number of experiments and are used to create statistically designed experiments, estimate coefficients, predict the experimental response and validate the model [30]. The second-order quadratic model was chosen for the process in the optimisation, as given by Equation (5), to identify the presence of parameter interactions:

(5) $$Y=\beta_{0}+\sum \beta_{i}x_{i}+{\left(\sum \;\beta_{ii}x_{i}\right)}^{2}+\sum \beta_{ij}x_{i}x_{j}$$

where *β*_0_ is the regression coefficient term, *β_i_* is the linear coefficient term, *β_ii_* is the quadratic coefficient term, *β_ij_* is the interaction coefficient term and *x_i_* as well as *x_j_* are the levels of the independent factors.

The use of a CCD in RSM generates an assessment of the overall significance of the model (*p*-value), the coefficient of determination, adequate precision and the coefficient of variance through the use of ANOVA. Although a key element of a CCD is in its determination of significant interactions between factors, it also identifies the significance of each individual factor [31].

Model verification was performed based on the point prediction in the post-analysis of the process, by comparing the predicted and experimentally determined values. Table 2 gives the experimental design for the validation of biosurfactant production by WCO and PCO cultures.

### 2.6. Statistical Analyses

All experiments were carried out in triplicate and variation was indicated by the standard error of the mean (SEM). One-way analysis of variance (ANOVA) was used to analyse the data, and where significant was followed by post hoc pairwise Tukey’s tests, carried out using GraphPad InStat (version 3.1). *p* < 0.05 was accepted as statistically significant.

## 3. Results

### 3.1. Degradation Kinetics Modelling

**Figure 1:**
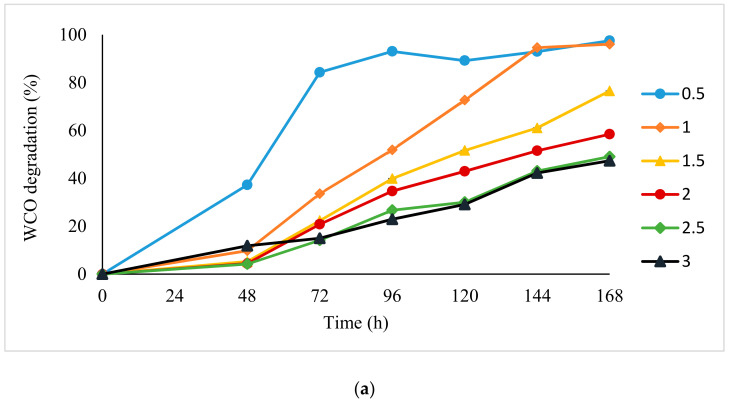

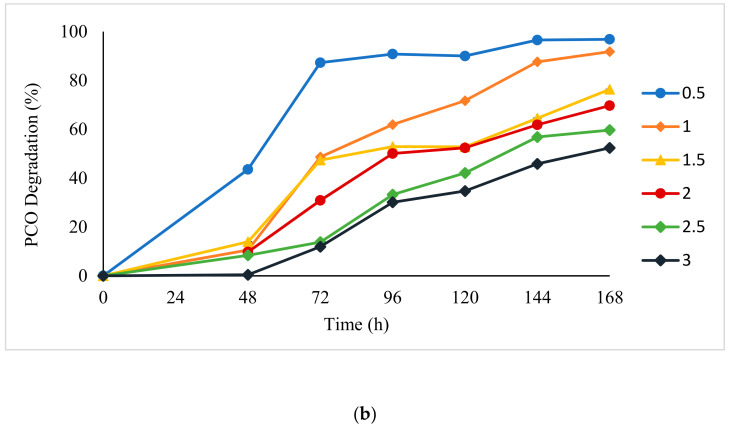
Effect of initial substrate concentration on degradation of (**a**) WCO and (**b**) PCO by BS14 bacterial consortium. Error bars represent mean ± standard error (SEM) and are contained within the points.

WCO and PCO degradation was assessed over 7 days in cultures with different initial substrate concentrations (0.5% to 3.0%). For both WCO and PCO, the lowest initial concentration of the substrate yielded high degradation percentages over the 168 h culture period. The highest levels of degradation achieved were about 93% and 96%, respectively, at 0.5% initial substrate concentration. The percentage of WCO and PCO degradation reduced rapidly at initial concentrations above 1.5% (Figure 1), although the absolute amount of WCO and PCO degraded at 3% initial concentration was still approximately three times greater than that at 0.5% initial concentration.

**Figure 2:**
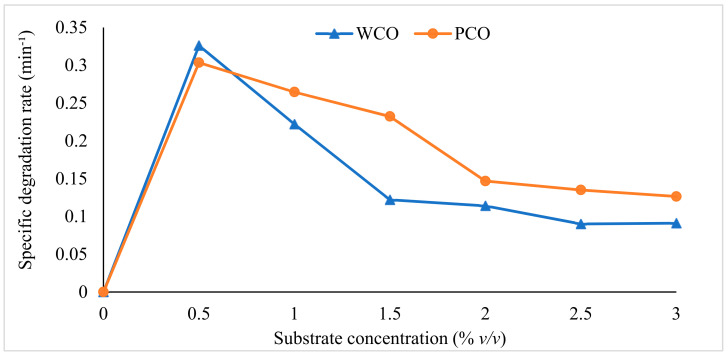
Specific degradation rates of WCO and PCO with increasing initial substrate concentration.

The relationship between the specific degradation rate and the initial substrate concentration is shown in Figure 2. As noted above, the highest degradation rates of WCO and PCO were achieved at the lowest initial substrate concentration (0.5%), with the rate then decreasing as the initial substrate concentration increased. The WCO degradation rate decreased more rapidly with an increasing substrate concentration than did that of PCO.

**Figure 3:**
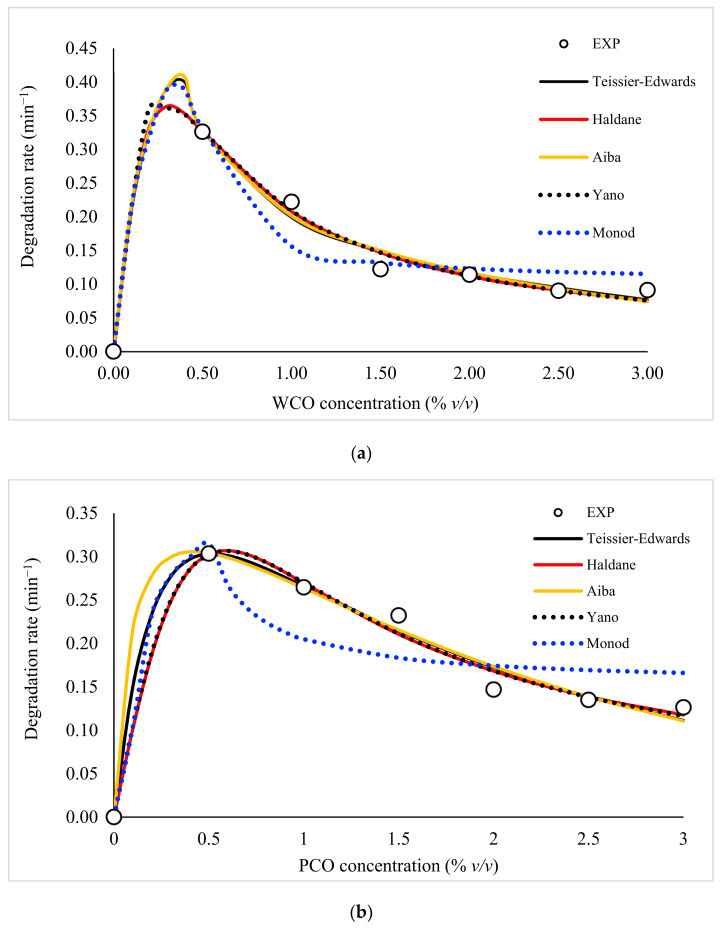
Kinetic modelling of (**a**) WCO and (**b**) PCO degradation using a range of standard non-linear models.

The relationship between degradation rate and initial substrate concentration was modelled using a range of standard non-linear models (Figure 3 and Supplementary Materials Table S1). The Haldane and Yano models provided the mathematical best fit to the experimental data; both models assume substrate inhibition at higher concentrations. Table 3 shows the statistical analysis across all the models tested, again identifying that the Haldane model provided the mathematical best fit for both WCO and PCO degradation. However, the fits of the Haldane and Yano models were very similar.

The half-saturation constant (*K_s_*) and inhibition constant (*K_i_*) in the Haldane model for WCO biodegradation were 5.14% and 0.019%, respectively. Meanwhile, in the biodegradation of PCO, the *K_s_* value was 16.65% while the *K_i_* value was 0.021%. As these values were obtained from the regressed equation, the maximum concentration of substrate tolerated *(S_m_*) and the actual maximum degradation rate (*μ_max_*) could be calculated. The *S_m_* value for WCO (0.31%) was somewhat lower than that for PCO (0.59%), while the *μ_max_* value for WCO was slightly higher (0.37 min^−1^) than that for PCO (0.31 min^−1^).

### 3.2. Preliminary Screening

The ability of bacterial consortium BS14 to produce biosurfactants during WCO and PCO biodegradation was next assessed. Preliminary screening for biosurfactant production utilised the haemolytic test, a microbial adhesion to hydrocarbons assay, the oil-spreading test, the drop-collapse test and emulsification index (E_24_).

#### 3.2.1. Haemolytic Test

**Figure 4:**
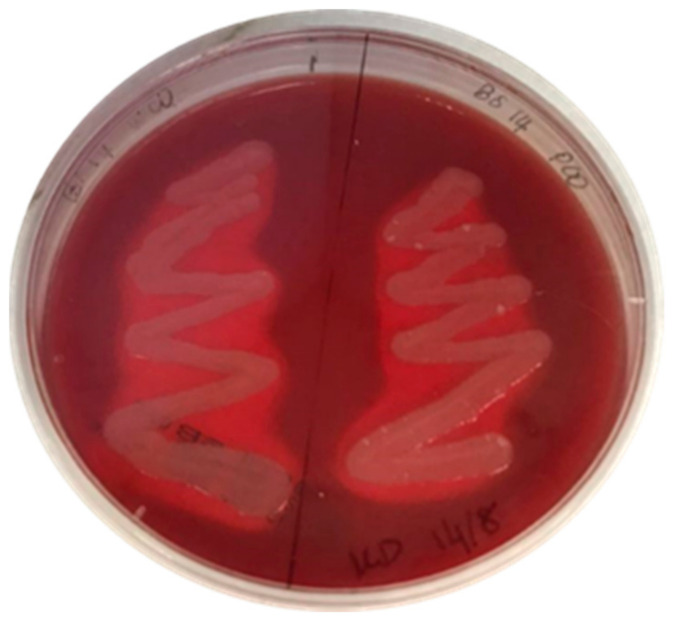
BS14 consortium inoculated from WCO (left) and PCO (right) media demonstrating β-haemolytic activity on a blood agar plate after a 2 days incubation.

The ability of the BS14 consortium to produce biosurfactants was tested on blood agar. Inocula from both WCO and PCO media showed positive results (Figure 4). Clear zones indicating β-haemolytic activity can be seen surrounding the bacterial growth, indicating the presence of biosurfactant-producing bacteria.

#### 3.2.2. Microbial Adhesion to Hydrocarbons (MATH) Assay

**Figure 5:**
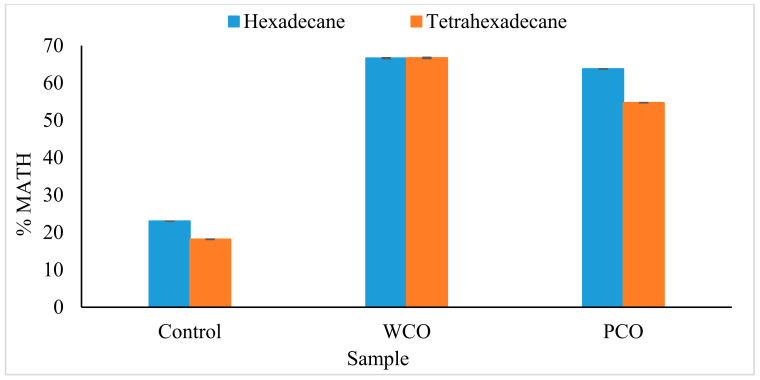
Microbial adhesion of BS14 bacterial consortium to two different hydrocarbon substrates. Error bars represent mean ± standard error (SEM).

Organic solvents were used in this assay as the hydrocarbon substrates. The assay indicated that WCO and PCO samples were characterised by >50% microbial adhesion to hexadecane and tetrahexadecane (Figure 5).

#### 3.2.3. Oil-Spreading and Drop-Collapse Test

Both these assays are qualitative tests that determine the presence of biosurfactants in the media. Oil displacement was clear in the oil-spreading test for both WCO and PCO media, with magnitudes of 23.00 and 31.17 mm diameter, respectively. The supernatant’s ability to collapse a hydrocarbon droplet also showed positive results (Table 4).

#### 3.2.4. Emulsification Index (E_24_)

**Figure 6:**
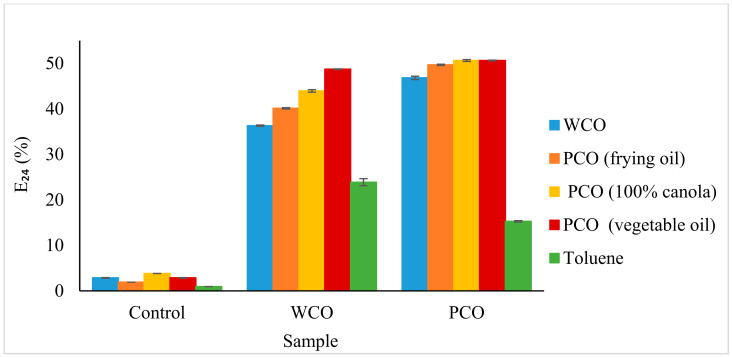
Emulsification index after a 24 h incubation (E_24_) for both cell-free WCO and PCO MSM culture supernatants. Error bars represent mean ± standard error (SEM).

The emulsification test is an indirect method used to screen for biosurfactant production. Figure 6 shows the ability of cell-free WCO and PCO MSM culture supernatants to emulsify different types of cooking oil as hydrocarbon substrates. The biosurfactants in the supernatants emulsified >40% of the cooking oils and >10% of the toluene organic solvent. Even though the E_24_ for toluene was lower than those of other hydrocarbons, the capability of the biosurfactant to emulsify this organic solvent was greater than that of the negative control.

### 3.3. Measurement of Biosurfactant Production

**Figure 7:**
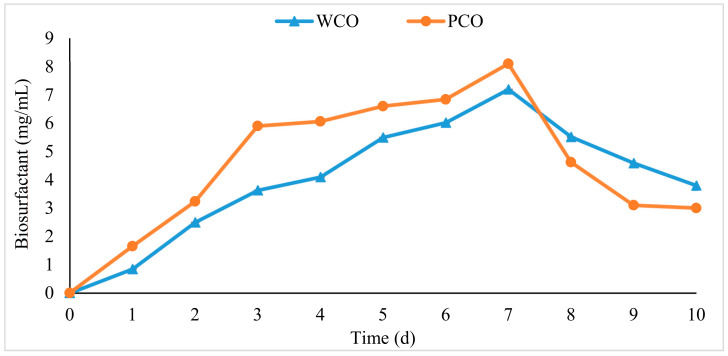
Biosurfactant production over a 10 days incubation by BS14 bacterial consortium. Error bars represent mean ± standard error (SEM) and are contained within the points.

The production of biosurfactants in WCO and PCO media was measured over 10 days of incubation. The highest production of biosurfactant was measured on day seven, with 7 and 8 mg/mL being produced in WCO and PCO media, respectively (Figure 7). The amount of biosurfactant produced in both cultures dropped sharply after day seven.

### 3.4. Optimisation of Biosurfactant Production

Table 5 and Table 6 display the analysis of variance (ANOVA) for PBD for biosurfactant production using WCO or PCO as a substrate, respectively. The factors with a significant influence on the degradation response were salinity (A), pH (B) and substrate concentration (C) for both WCO and PCO media.

All significant factors (A, B and D) were then further optimised using a CCD (Table 7). Twenty runs were generated from the software with the predicted and actual values for the biosurfactant production using either WCO or PCO as a substrate.

The second-order fitting equation’s coefficients were calculated by the software and the model acceptability was verified using ANOVA. The second-order polynomial coded equations are given as Equations (6) and (7), for WCO and PCO, respectively:

Y = −2634.60 + 47.40A + 721.46B + 18.58C − 19.07AC − 128.07A^2^ − 49.43B^2^ − 5.66C^2^ (6)

Y = −1.94 + 1.68BC − 49.54A^2^ + 0.098B^2^ − 4.87C^2^ (7)

The significant coefficients identified in WCO media included A, B, C, AC, A^2^, B^2^ and C^2^, while in PCO media these included BC, A^2^, B^2^ and C^2^.

In CCDs, different types of adequacy of the model were tested, including linear, 2FI, quadratic and cubic. For both WCO and PCO, the statistical model showed a high value of R^2^ (>0.9) in the quadratic (Table 8 and Table 9) and cubic models. The R^2^ values generated in the cubic model were somewhat higher than the quadratic model: 0.971 (WCO) and 0.973 (PCO). Nevertheless, the model suggested by the software was the quadratic model for both WCO and PCO.

ANOVA for both CCDs in biosurfactant production using WCO and PCO showed that the model designs were highly significant (Table 8 and Table 9). The importance of the single factors in CCDs can be inferred from the F values calculated in the design. Table 8 shows that *F* _WCO concentration_ > *F* _pH_ > *F* _NaCl_, suggesting that WCO concentration was the most influential factor towards biosurfactant production when using WCO as a substrate. When PCO was used as substrate, pH was the most influential factor, followed by NaCl and then PCO concentration (Table 9). Significant interactions between any two factors during the optimisation process are also identified through CCDs. Significant interactions were identified between salinity (A) and WCO concentration (C) in biosurfactant production using WCO as a substrate, and between pH (B) and PCO concentration (C) using PCO as a substrate.

**Figure 8:**
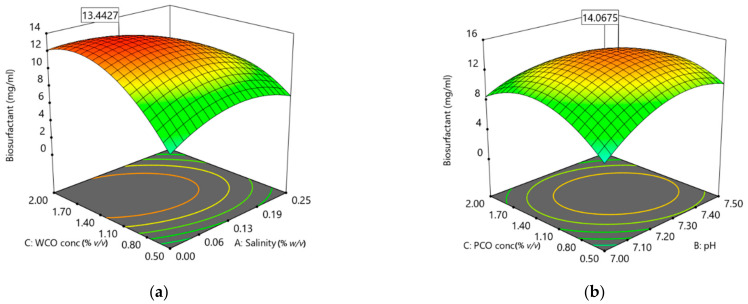
Three-dimensional (3D) response surface plots for the significant factors identified in CCD for biosurfactant production using WCO, (**a**): salinity and WCO concentration; and PCO (**b**): pH and PCO concentration.

As illustrated in the 3D response surface plots (Figure 8), high concentrations of WCO (1.40 to 1.70%) with low salinity (0.06 to 0.13%) were predicted to lead to high biosurfactant production (13.44 mg/mL). Low WCO and high salt concentration decreased the ability of BS14 bacterial consortium to produce biosurfactants. The optimum conditions for biosurfactant production in PCO media were predicted to be a medium pH, 7.20–7.30, and an initial PCO substrate concentration of 1.10–1.14% (*w*/*v*), yielding a highest biosurfactant production of 14.07 mg/mL.

**Figure 9:**
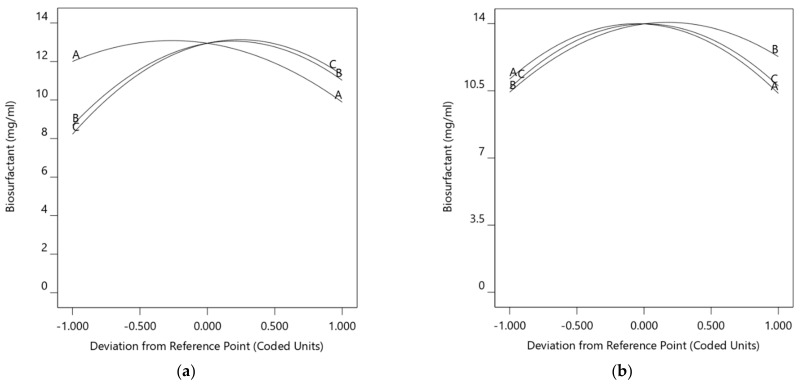
Perturbation plots for biosurfactant production using (**a**) WCO and (**b**) PCO as substrate.

The perturbation plots (Figure 9) illustrate the comparative effects of all factors on biosurfactant production. Although ANOVA only identified one significant interaction for both conditions, all factors still have a major contribution to biosurfactant production: salinity (A), pH (B) and substrate concentration (C). The curvature of the perturbation plot factors shows that the response was sensitive and influenced by all the factors.

The results of the responses in CCDs were validated experimentally using the specific conditions generated by the software. Table 10 shows the results for WCO and PCO media, with no significant difference between the actual and predicted values in either case, validating the model.

## 4. Discussion

### 4.1. Mathematical Modelling

The BS14 Antarctic bacterial consortium was confirmed to produce biosurfactants in the presence of WCO or PCO as a substrate, showing good ability to degrade both WCO and PCO. To our knowledge, there have been no previous studies modelling the biodegradation kinetics of WCO and PCO using bacterial consortia isolated from Antarctic soils, and only one study addressing the kinetics of bacterial growth by Antarctic bacteria has been reported [14].

The relationship between degradation rate and initial concentration of substrate is important for large-scale bioremediation applications. Our data indicated that the BS14 bacterial consortium achieved better degradation of PCO than WCO. Bacteria generally cannot degrade highly concentrated oil due to its properties and toxicity. Cooking oil changes in composition and properties when heated above a certain temperature [32]. For example, the oxidative stability of canola oil can lead to peroxide formation in three days under accelerated conditions at 60–65 °C [33]. Lipid oxidation during cooking can lead to the production of aldehydes, epoxides, hydroxyketones and dicarboxylic compounds. These compounds can react with amino acids to produce the carcinogenic compound acrylamide [34].

The complex structure of the PCO molecule could also inhibit the bacterial community from degrading the PCO at high concentrations. PCO has a homogenous fatty acid composition, with 95% unsaturated and saturated fatty acids, including oleic acid, linoleic acid, linolenic acid, stearic acids and palmitic acids [35,36]. Generally, canola oil has 18 carbon fatty acids in its total unsaturated fatty acids and antioxidants. The biodegradability of long-chain fatty acids decreases with an increasing carbon chain length and a decreasing degree of unsaturation [37]. Several studies have confirmed low degradation at high concentrations of cooking oil [38,39,40].

Of the different kinetic models examined here, the Haldane, Aiba, Yano and Teissier–Edwards models predicted inhibition at high substrate concentrations. The Monod model assumed a critical inhibitor concentration, above which bacterial cells cannot grow and function. The Haldane model is widely used in kinetic studies due to its ability to integrate substrate and growth inhibition constants as well as its simplicity [40]. Generally, the Haldane model is considered an extension of the Monod model, introducing a third constant parameter known as the inhibition constant (*K_i_*). A previous study on phenol degradation and the growth kinetics of *P. aeruginosa* demonstrated that the best fit was provided by the Haldane model [41], as was also found using *P. putida* in the biodegradation of phenol [42]. Free cyanide removal by *Acinetobacter courvalinii* was also best-fitted using the Haldane model [43]. Wang et al. [44] similarly reported a good fit using the Haldane model in the removal of ammonia/nitrate using *Acinetobacter* sp.

The BS14 bacterial consortium could function and degrade 0.5 and 1% initial concentrations of WCO and PCO, respectively. However, more than 1.5% initial concentration of either media reduced the percentage degradation of the initial concentration achieved. The mathematical best fit was provided by the Haldane model, with high substrate concentrations reducing the rate of activity [45]. The practical application of such models lies in the assessment of product formation kinetics, and in their utility in monitoring biological processes in the real world [10].

### 4.2. Preliminary Test on Biosurfactant Production

The preliminary test on biosurfactant production in this study was used to confirm the BS14 consortium’s ability to produce biosurfactants. The haemolytic test has long been used in the preliminary screening of biosurfactant-producing microorganisms [26,28,46,47]. Surfactants are generally able to lyse the erythrocytes [48] by altering the structure of membrane lipids and proteins. cell lysis is caused by osmotic and solubilisation haemolysis [49,50]. of 89 strains collected from seawater samples, 24% showed a positive result in the haemolysis test, with 10% of these showing α-haemolytic activity and 24% showing β-haemolytic activity [51]. Nonetheless, the haemolytic test has a limitation in that lytic enzymes may also be present in the consortium, which can also cause clearing zones [52]. As a result, this method is only recommended as a preliminary screening step [53], which should be supplemented by other techniques, such as those described below.

The hydrophobicity of the microbial cell surface can be determined using a MATH assay, which is a simple and rapid method [54]. Microorganisms can adsorb on the surface of an organic pollutant by increasing the hydrophobicity of their cell surface, which takes place through the remodelling of the cell’s outer layers [55]. The positive results obtained here confirmed the affinity of cells towards the hydrocarbons. The process of cell adherence to hydrophobic compounds is considered an indirect method to screen bacteria for the production of biosurfactants. This is because the cells are able to attach themselves to the hydrocarbons by producing surface-active compounds called biosurfactants [56]. The effectiveness of this method has been proven by studies in which W-28 and ISL-01 bacterial isolates from hydrocarbon-rich soil and effluent showed a high hydrophobicity value of 58% [57], *Pseudomonas aeruginosa* MS3 isolated from oil-contaminated marine sediments showed 50% hydrophobicity [58] and *Bacillus subtilis* isolated from a pharmaceutical effluent sample was able to adhere to kerosene up to 58% [59].

The size of the clearing zone in the oil spreading test indicates the concentration of the biosurfactant solution [60] and that the interfacial tension between the hydrophobic part and biosurfactant is reduced [61]. Previously, *Streptomyces* sp., *Microbacterium* sp., *Rhodococcus* sp., *Arthrobacter* sp. and *Bacillus subtilis* have been shown to produce clearing zone diameters of 33, 23, 16, 29 and 35 mm, respectively, using this test [24]. These bacterial strains were also reported to give positive results in the drop-collapse test. A study on lactic acid bacteria including *Lactobacillus fermentum* strain SHU6343, *Pediococcus dextrinicus* strain SHU1593, *L. plantarum* strain SHU3455, *L. rhamnosus* strain SHU1904 and *L. dextrinicus* strain SHU68 confirmed positive results in both oil-spreading and drop-collapse tests in the screening for the production of biosurfactants [62]. Both assays are widely used for screening purposes because they are rapid, simple to perform, do not require specialised equipment and require a small number of samples [63].

The E_24_ test is a well-known method for the screening of microorganisms that can produce biosurfactants, because one of the key characteristics of biosurfactants is to emulsify hydrocarbons. Amodu et al. [64] reported that the biosurfactants produced by *Bacillus licheniformis* strain STK and *B. subtilis* strain STK 02 can emulsify better in oils such as diesel and lubricant oil (60 to 90%) compared to the organic solvent hydrocarbon, kerosene (< 20%). In contrast, the biosurfactant produced by *Rhodococcus erythropolis* strain AQ5-07 showed higher E_24_ in organic solvents rather than in diesel oil, with hexane, hexadecane and tetrahexadecane being emulsified more than 80% while only 60% of diesel was emulsified [13]. *Arthrobacter* spp. strains AQ5-05 and AQ5-06 from Antarctica also exhibited high E_24_ values in hexadecane (60% and 67%, respectively), though there was only a thin layer for both strains when using diesel [65].

### 4.3. Biosurfactant Production

In our study, the production of biosurfactants was measured over 10 days in WCO and PCO media, with the highest production being observed on day seven for both substrates, at 7 and 8 mg/mL, respectively. Different microorganisms might require different incubation times to produce the maximum amount of biosurfactant. *Virgibacillus salaries* was reported to require between 3 to 6 days to produce 2.8 to 2.9 g/L of biosurfactant [66], while the marine bacterium *Nocardiopsis* sp. strain B4 required 7 to 9 days to yield its maximum amount of biosurfactant [67].

The highest biosurfactant production phase can also be estimated by observing the pattern of bacterial growth in the presence of the substrate. In general, the biosurfactant starts to be produced during the stationary phase and continues until the cell biomass starts to decrease or the death phase is reached. However, in some cases, the biosurfactant itself may be utilised by the bacteria during the survival–starvation state [68].

The important factors in biosurfactant production were optimised using response surface methodology. The factors were screened first using PBD to eliminate non-significant factors. In CCDs, the use of a polynomial model in this study allowed the prediction reliability of the model to be improved, eliminating non-significant predictor factors and reducing model error [69].

The optimum conditions for the bacteria to produce high biosurfactant concentrations in WCO and PCO media can be analysed using 3D contour plots. Bacteria such as *Acinetobacter* sp., *P. aeruginosa*, *B. subtilis* and *B. licheniformis* show the greatest production in low-salinity conditions [32,70,71], similar to the current study. *P. aeruginosa* strain UKMP14T and *Klebsiella* sp. strain KOD36 also required 1% initial substrate concentration to yield a high percentage of surface tension reduction and E_24_ activities [72,73]. In some cases, salinity can reduce soil microorganism activity due to osmotic stress and the presence of toxic ion concentrations. Soluble salts may increase the osmotic potential of water in the soil, drawing water out of the cells and even eventually killing microorganisms through plasmolysis [74]. Numerous studies have shown that salinity can lead to reduced microbial biomass as well as changing the microbial community structure and activity [75,76,77,78]. A similar study, Amodu et al. [62], reported that biosurfactant activity was determined through surface tension reduction. The central point of the CCD based on the contour plot identifies the optimum point of the response. In the current study this was between 6.5 to 8.0 for pH and 4 to 8% for initial substrate concentration. *B. subtilis* strain UKMP-4M5 can similarly produce a biosurfactant and the optimum pH at pH 6 to 8 yielded 32 to 34 mN/m surface tension [60]. In another study, biosurfactant production in *Lactococcus lactis* strain CECT-4434 was tested in two conditions, with or without pH control. With pH control, a high production of biosurfactant was measured through surface tension analysis [72]. This shows the importance of studying pH in environmental microbiology [79]. Variation in environmental pH can also affect metabolic activity in natural communities, as different microorganisms prefer different pH conditions. Variation in pH can interfere with microbial metabolism and modulate thermodynamic and redox reactions [80]. The slightly alkaline conditions in the current study contributed to the optimisation of biosurfactant production.

The yield of the biosurfactant generally increases with the concentration of substrate [81]. Here, low biosurfactant yield was obtained at low concentration of WCO during the optimisation process. Other studies have reported similar results, such as the ability of *B. subtilis* strains ATCC 21332 and MSH1 to produce up to 450 and 200 mg/L of biosurfactant, respectively, at a high concentration of glucose (around 30 g/L) [82]. However, in certain cases high substrate concentration can reduce biosurfactant production [83]. In such cases, the high substrate concentration could inhibit the bacterial growth and metabolism.

Although biosurfactants have higher surface activity with high tolerance to various factors and can withstand up to extreme conditions (acidity or basicity of an aqueous solution, temperature, salt concentration, ionic strength and demulsifying as well as emulsifying ability) [84], several important factors need to be optimised in order for bacteria to produce biosurfactants during the biodegradation of oil, as carried out in this study. Microorganisms increase the bioavailability of potentially biodegradable nutrients, such as oil, by producing biosurfactants [85]. Biosurfactants influence microbial cell surface properties, causing significant alterations of surface hydrophobicity and surface functional groups. The cell surface hydrophobicity influences the tendency of cells to adhere to the hydrophobic part, which is commonly analysed through a MATH assay. Interactions of cells with their environment are also influenced by carboxyl, phosphate and amino functional groups present on the cell surface. Changes in cell surface characteristics appear to be correlated with cell adhesion to pollutants [86].

## 5. Conclusions

The mathematically best-fitting kinetic model for the biodegradation of WCO and PCO using the BS14 bacterial consortium was the Haldane model, although several models gave closely similar fits to the experimental data. The consortium also proved to be a strong biosurfactant producer when using WCO and PCO as substrates. The biosurfactant produced gave positive results in the haemolytic test, oil-spreading test, drop-collapse test and had a high percentage of hydrophobicity and emulsification index. The critical factors in biosurfactant production included salinity, pH, temperature and initial substrate concentration, which were optimised to produce the highest yield. This study provides important information for the development of bioremediation protocols for the degradation of oils, such as canola oil, in the Antarctic. The presence of biosurfactants in the bioremediation of oil could enhance the rate of biodegradation activity efficiently, as biosurfactants could increase the hydrocarbons’ mobility and bioavailability. Thus, the biosurfactant produced during the degradation process has potential for use in other applications such as the in situ or ex situ bioremediation of other hydrocarbons, including diesel and petroleum-derived oils.

## Figures and Tables

**Table 1:** Plackett–Burman experimental design for biosurfactant production by WCO and PCO using BS14 bacterial consortium.

Run	Standard Deviation	A	B	C	D
1	3	0.00	7.50	15.00	0.50
2	6	0.25	7.50	15.00	0.50
3	5	0.25	7.50	10.00	2.00
4	2	0.25	7.50	10.00	2.00
5	10	0.25	7.00	10.00	0.50
6	4	0.25	7.00	15.00	2.00
7	8	0.00	7.00	15.00	2.00
8	11	0.00	7.50	10.00	0.50
9	7	0.00	7.50	15.00	2.00
10	9	0.00	7.00	10.00	2.00
11	12	0.00	7.00	10.00	0.50
12	1	0.25	7.00	15.00	0.50

WCO: waste canola oil, PCO: pure canola oil, BS14: coded Antarctic bacterial consortium, A: salinity (% *w*/*v*); B: pH; C: temperature (°C); and D: WCO/PCO initial concentration (% *v*/*v*).

**Table 2:** Experimental design for the validation of biosurfactant production by BS14 bacterial consortium.

Substrate	A	B	C	Predicted (mg/mL)
WCO	0.00	7.35	2.0	12.65
PCO	0.05	7.27	1.76	10.94

A: salinity (% *w*/*v*), B: pH, C: WCO/PCO initial concentration (% *v*/*v*).

**Table 3:** Statistical analysis of different standard models applied to WCO and PCO degradation kinetics.

Model	R^2^	Adj R^2^	DF	RMSE	AICc	SSE	AF	BF
WCO								
Haldane	0.985	0.992	4	0.017	−54.814	0.001	0.999	0.999
Yano	0.985	0.992	3	0.019	−47.758	0.001	0.999	0.999
Teissier–Edwards	0.978	0.989	4	0.030	−45.251	0.004	1.000	1.000
Aiba	0.977	0.988	4	0.020	−51.869	0.002	0.999	0.999
Monod	0.910	0.954	5	0.035	−46.654	0.006	1.000	1.000
PCO								
Haldane	0.983	0.991	4	0.016	−54.821	0.001	0.999	0.999
Yano	0.984	0.992	3	0.019	−47.821	0.001	0.999	0.999
Teissier–Edwards	0.981	0.991	4	0.017	−53.802	0.001	0.999	0.999
Aiba	0.981	0.990	4	0.016	−53.553	0.001	0.999	0.999

R^2^: coefficient determination, Adj R^2^: Adjacent coefficient, DF: degree of freedom, RMSE: root mean square error, AICc: Akaike information criterion, SSE: sum of squared estimate of errors, AF: accuracy factor, BF: bias factor.

**Table 4:** Oil spreading and drop collapse test results achieved by BS14 bacterial consortium in WCO and PCO media.

Sample	Diameter (mm)	
	Oil Spreading Test	Drop Collapse Test
Control	5.67	6.67
WCO	23.00	9.33
PCO	31.17	9.00

**Table 5:** Analysis of variance (ANOVA) for Plackett-Burman design for biosurfactant production using WCO as a substrate.

Source	Sum of Squares	DF	*F* Value	Prob > *F*
Model	82.83	4	17.06	0.0078 **
A	9.82	1	14.16	0.0197 *
B	8.43	1	12.16	0.0252 *
C	2.21	1	3.18	0.1491
D	28.13	1	40.56	0.0031 **
Residual	2.77	7		
Cor Total	85.61	11		
*R*-squared	0.968		Pred *R*-squared	0.745
Adj *R*-squared	0.911		Adeq Precision	11.58

A: salinity (% *w*/*v*), B: pH, C: temperature (°C), D: WCO concentration (% *v*/*v*). * *p* < 0.05, ** *p* < 0.01.

**Table 6:** Analysis of variance (ANOVA) for Plackett-Burman design for biosurfactant production using PCO as a substrate.

Source	Sum of Squares	DF	*F* Value	Prob > *F*
Model	54.83	4	32.72	0.0023 **
A	11.33	1	47.34	0.0023 **
B	1.96	1	8.17	0.0460 *
C	0.54	1	2.25	0.2082
D	7.68	1	32.07	0.0048 **
Residual	0.96	7		
Cor Total	55.79	11		
*R*-squared	0.993		Pred *R*-squared	0.861
Adj *R*-squared	0.953		Adeq Precision	18.02

A: salinity (% *w*/*v*), B: pH, C: temperature (°C), D: PCO concentration (% *v*/*v*). * *p* < 0.05, ** *p* < 0.01.

**Table 7:** Central composite experimental design for biosurfactant production by BS14 bacterial consortium using either WCO or PCO as substrate.

Run	Standard Deviation	A	B	C	WCO		PCO	
					Actual	Predicted	Actual	Predicted
1	17	0.13	7.25	1.25	13.80	12.95	6.73	5.29
2	8	0.25	7.50	2.00	3.02	4.06	13.50	13.80
3	13	0.13	7.25	−0.01	1.31	1.37	12.46	13.98
4	18	0.13	7.25	1.25	10.36	12.99	1.71	4.55
5	20	0.13	7.25	1.25	11.73	12.93	3.50	4.17
6	2	0.25	7.00	0.50	2.43	4.04	7.27	6.81
7	3	0.00	7.50	0.50	3.15	4.04	4.40	4.98
8	12	0.13	7.67	1.25	5.44	6.19	5.40	6.31
9	11	0.13	6.83	1.25	3.27	2.24	13.50	13.90
10	4	0.25	7.50	0.50	5.27	3.69	14.30	14.00
11	14	0.13	7.25	2.51	6.88	6.53	3.95	4.73
12	15	0.13	7.25	1.25	13.87	12.90	4.23	4.15
13	19	0.13	7.25	1.25	13.96	13.00	14.76	14.99
14	7	0.00	7.50	2.00	12.97	11.57	3.83	2.92
15	10	0.34	7.25	1.25	5.64	5.51	4.32	4.31
16	6	0.25	7.00	2.00	3.35	2.67	3.77	1.99
17	9	−0.09	7.25	1.25	9.23	9.06	4.69	5.43
18	1	0.00	7.00	0.50	1.6	0.77	10.69	8.78
19	16	0.13	7.25	1.25	13.91	13.50	6.20	4.71
20	5	0.00	7.00	2.00	4.75	6.54	15.63	14.99

A: salinity (% *w*/*v*), B: pH, C: WCO/PCO concentration (% *v*/*v*).

**Table 8:** Analysis of variance (ANOVA) for central composite design (CCD) for biosurfactant production using WCO as substrate.

Source	Sum of Squares	DF	*F* Value	Prob > *F*
Model	54.83	9	16.08	<0.0001 ***
A	11.33	1	5.69	0.0383 *
B	1.96	1	6.94	0.0249 *
C	0.54	1	12.05	0.0060 **
AB	7.68	1	2.46	0.1481
AC	12.47	1	9.54	0.0115 *
BC	25.50	1	0.57	0.4670
A^2^	9.45	1	21.54	0.0009 ***
B^2^	0.96	1	51.34	<0.0001 ***
C^2^	0.55	1	54.45	<0.0001 ***
Residual	0.41	10		
Lack of Fit	55.79	5	1.28	0.3962
Pure Error	54.83	5		
Cor Total	11.33	19		
*R*-squared	0.935		Pred *R*-squared	0.638
Adj *R*-squared	0.877		Adeq Precision	10.521

A: salinity (% *w*/*v*), B: pH, C: WCO concentration (% *v*/*v*). * *p* < 0.05, ** *p* < 0.01, *** *p* < 0.001.

**Table 9:** Analysis of variance (ANOVA) for central composite data design (CCD) for biosurfactant production using PCO as substrate.

Source	Sum of Squares	DF	*F* Value	Prob > *F*
Model	377.91	9	14.81	0.0001 ***
A	1.91	1	0.67	0.4306
B	11.72	1	4.13	0.0694
C	0.06	1	0.02	0.8895
AB	3.30	1	1.17	0.3058
AC	4.26	1	1.50	0.2482
BC	14.84	1	5.24	0.0452 *
A^2^	152.00	1	53.61	<0.0001 ***
B^2^	99.82	1	35.20	0.0001 ***
C^2^	156.55	1	55.21	<0.0001 ***
Residual	28.35	10		
Lack of Fit	22.16	5	3.57	0.0942
Pure Error	6.20	5		
Cor Total	406.26	19		
*R*-squared	0.930		Pred *R*-squared	0.558
Adj *R*-squared	0.867		Adeq Precision	10.071

A: salinity (% *w*/*v*), B: pH, C: PCO concentration (% *v*/*v*). * *p* < 0.05, *** *p* < 0.001.

**Table 10:** Validation of predicted response surface model.

Substrate	Biosurfactant (mg/mL)		*p* Value
	Expected Value	Actual Value	
WCO	12.65	11.97	0.787
PCO	10.94	10.78	0.954

## Data Availability

The datasets generated for this study are available on request to the corresponding author.

## Supplementary Materials

The following are available online at https://www.mdpi.com/article/10.3390/foods10112801/s1, Table S1: Kinetic modelling data on WCO and PCO degradation.
