# Supplementary material for: Mathematical Modelling of Canola Oil Biodegradation and Optimisation of Biosurfactant Production by an Antarctic Bacterial Consortium Using Response Surface Methodology
Source: Foods. 2021 Nov 14;10(11):2801. doi: 10.3390/foods10112801 (PMC8621366; doi:10.3390/foods10112801)
Supplement: Supplementary file 1 [file foods-10-02801-s001.zip › foods-1380067-supplementary.pdf]

Table S1. Kinetic modelling data on WCO and PCO degradation

Oil concentration (%)	Degradation rate (min^{-1})					
WCO	EXP	Haldane	Yano	Teissier	Aiba	Monod
0.00	0.00	0.000	0.000	0.000	0.000	0.000
0.10	NA	0.216	0.211	0.211	0.216	0.214
0.20	NA	0.333	0.361	0.328	0.328	0.318
0.30	NA	0.364	0.361	0.391	0.391	0.389
0.40	NA	0.353	0.352	0.399	0.408	0.389
0.50	0.326	0.327	0.327	0.329	0.328	0.331
1.00	0.222	0.208	0.209	0.199	0.201	0.156
1.50	0.122	0.147	0.147	0.149	0.150	0.132
2.00	0.114	0.112	0.112	0.117	0.117	0.123
2.50	0.090	0.091	0.090	0.095	0.093	0.118
3.00	0.091	0.076	0.075	0.077	0.074	0.115
PCO						
0.00	0.00	0.000	0.000	0.000	0.000	0.000
0.10	NA	0.102	0.105	0.151	0.217	0.105
0.20	NA	0.188	0.190	0.234	0.278	0.234
0.30	NA	0.248	0.250	0.277	0.299	0.278
0.40	NA	0.285	0.285	0.297	0.305	0.299
0.50	0.303	0.303	0.302	0.303	0.304	0.315
1.00	0.264	0.269	0.270	0.266	0.263	0.205
1.50	0.232	0.211	0.211	0.215	0.215	0.183
2.00	0.147	0.168	0.168	0.173	0.173	0.174
2.50	0.135	0.139	0.138	0.139	0.139	0.169
3.00	0.126	0.118	0.116	0.111	0.111	0.166

EXP: experimental values, NA: not available
